# High Temperature Limits of Survival and Oviposition of *Phormia regina* (Meigen) and *Lucilia sericata* (Meigen)

**DOI:** 10.3390/insects13110991

**Published:** 2022-10-28

**Authors:** Michael A. Monzon, Lauren M. Weidner, Travis W. Rusch, Selen Nehrozoglu, George Hamilton

**Affiliations:** 1 Department of Entomology, Rutgers, The State University of New Jersey, New Brunswick, NJ 08901, USA; 2 School of Mathematical and Natural Sciences, New College of Interdisciplinary Arts and Sciences, Arizona State University, Glendale, AZ 85306, USA; 3 School of Life Sciences, Arizona State University, Tempe, AZ 85281, USA; 4 Animal and Plant Health Inspection Service (APHIS)—Plant Protection and Quarantine (PPQ), United Stated Department of Agriculture (USDA), Linden, NJ 07036, USA

**Keywords:** thermal biology, survivorship, oviposition, mortality, fecundity

## Abstract

**Simple Summary:**

These experiments were conducted to investigate the impact of high temperatures on survival and egg laying of two forensically important blow flies, *Phormia regina* (Meigen) and *Lucilia sericata* (Meigen) (Diptera: Calliphoridae). Earlier experiments with closely related species indicated egg laying will stop below lethal temperatures. However, these data had not been collected for blow fly populations in the Mid-Atlantic United States. Upper and lower treatment temperatures were based on previous longevity studies of *P. regina.* Objective 1 was to determine the likely temperatures that resulted in 50% and 0% survival for both species. Survival trials were completed at 37 °C, 41 °C, 42 °C, 43 °C, and 44 °C. Objective 2 investigated the number of eggs laid at 40 °C, 42 °C and 43 °C for both species. For each temperature trial, adult flies were held in an incubator for 24 h. Both *P. regina* and *L. sericata* were reduced to 50% survival at ~41 °C, with 0% survival occurring for both species by 44 °C. Similarly, as temperature increased, the number of eggs laid decreased. *Phormia regina* ceased laying eggs by 41 °C, while *L. sericata* laid viable eggs until 43 °C. This experiment supports earlier work indicating female blow flies likely stop egg laying at adult fly sub-lethal temperatures and have complex mechanisms to mitigate extreme temperatures.

**Abstract:**

The temperature dependent development rates of blow flies allow blow flies to be used as biological clocks in forensic death investigations. However, the upper thermal limits of adult survival and oviposition, both required for producing larvae, remains largely unknown. Therefore, in this study we examined the impact of a range of temperatures between 37 °C and 44 °C on the likelihood of survival and egg-laying behavior of two species of medicolegal forensic importance, *Lucilia sericata* (Meigen) and *Phormia regina* (Meigen) (Diptera: Calliphoridae). To quantify the upper temperature limits of survival, adult fly colonies were exposed to 37 °C, 41 °C, 42 °C, 43 °C, and 44 °C for 24 h. Similarly for oviposition trials, adults of both species were exposed to 40 °C, 42 °C, and 43 °C with *P. regina* oviposition also observed at 41 °C. Trials lasted for 24 h with oviposition substrate replenished at the 12 h mark. A yes/no determination on egg deposition was made, eggs were counted, and a yes/no determination was made on egg hatch. Survival did not differ by species (*p* = 0.096). Overall, survival decreased with increasing temperatures, with ~100% at 37 °C, ~50% at 41 °C, ~37% at 42 °C, ~15% at 43 °C and 0% at 44 °C. *Lucilia sericata* laid eggs capable of hatch up to 43 °C, while *Phormia regina* egg-hatch was observed up to 41 °C. These results indicate a greater thermal tolerance of adult survival than for egg deposition and successful egg hatch, which supports previous experiments indicating blow flies stop laying eggs at sub-lethal temperatures. Furthermore, these data indicate that adult blow flies may find remains at or near time of death but may delay egg deposition until temperatures drop below an acceptable threshold.

## 1. Introduction

Typical blow fly (Diptera: Calliphoridae) development begins when a female deposits an egg on a protein source, usually carrion, which hatches into a larva, also called a maggot. The resulting maggot undergoes three larval stages called instars. During the latter part of the third stage the maggot ceases feeding and will then pupate [1]. Following pupation, an adult emerges from the pupal casing and the life cycle begins again. However, the rate of this development is largely temperature dependent, which consequently allows blow flies to be used as evidence in death investigations to estimate the minimum post-mortem interval (i.e., minimum time since death, given certain assumptions) [2,3,4,5].

When studying how an organism’s physiology responds to high temperatures, thermal biologists have devised key metrics to be considered when observing a population of organisms experiencing maximum heat stress. The temperature at which all members of a population die is called the LT_Max_ (lethal temperature maxima), also referred to as LT_100_, while loss of effective locomotor function occurs is the biological critical thermal maxima (CT_max_) [6]. Typically, there is a temperature where approximately 50% of the members of a population can survive an extended period which is defined as the Upper Incipient Lethal Temperature (UILT) [7]. The range of temperatures between the UILT and the biological critical temperature maxima is deemed the zone of resistance [6]. Conducting experiments within an organism’s zone of resistance may indicate the success of behavioral and physiological processes (e.g., egg laying) in challenging conditions such as the onset of CT_max_. Modeling of genetic responses to thermal events demonstrated these sub-lethal scenarios likely shape thermal performance of the population in the long-term while incurring detriments to physiology (i.e., inability to lay eggs) on an individual level in the short term [8]. Periods of ≥ 24 h under experimental conditions have been used to explore thermal maximal tolerances in the Arctic charr *Salvelinus alpinus* (Linnaeus) (Salmoniformes: Salmonidae), and two *Drosophila* species (Diptera: Drosophilidae) [7,9]. In high temperature studies of marine tardigrades near Denmark, *Halobiotus crispae* (Kristensen) (Parachela: Hypsibiidae) in the active stage exhibited an LT_Max_ of 35.8 °C when treated for ~24 h [10]. While a population of the limnic boreo-alpine tardigrade species *Borealibius zetlandicus* (Murray) (Parachela: Hypsibiidae) reached LT_Max_ when treated at 37 °C for 24 h [11].

In North America and Europe, thermal biology studies of *Phormia regina* (Meigen) and *Lucilia sericata* (Meigen) have documented larval development from low to high temperatures and adult thermal biology at cooler temperatures [1,12,13,14,15]. Adult *P. regina* survival at high temperatures was briefly commented on in a study in Maryland where the researchers found adults survived for 15 days when exposed to 37.8 °C, but only for 1 h when exposed to 44.5 °C [16]. However, Prrish & Bickley [16] did not examine the maximum temperature *P. regina* could survive during extended periods of time or report on egg deposition during high temperature treatments.

More recently blow fly oviposition up to 40 °C has been documented for calliphorid populations in Canada and the United Kingdom. These studies focused on quantities of eggs laid, providing a basis for our study of quantifying the upper temperature limits for adult survival and oviposition [17,18,19]. Hans et al. [17] found calliphorids (*P. regina* and *L. sericata*) likely experience a temperature dependent peak (i.e., thermal optima) in eggs laid, above which the number of eggs deposited decreases with a continued temperature increase [17]. Similarly, high temperature experiments with the Texas populations of *Chrysomya rufifacies* (Macquart) (Diptera: Calliphoridae) and *Cochliomyia macellaria* (Fabricius) (Diptera: Calliphoridae) demonstrated that as temperature increased, the probability of survival decreased and the probability of knockdown increased [20,21]. This work revealed the importance of studying biological processes in both lethal and non-lethal upper temperatures of blow flies. Upper lethal temperatures provide information about where and when blow fly populations can persist, while upper non-lethal temperatures provide information as to where and when female blow flies can likely oviposit. Both conditions are vital for blow flies to colonize remains. This is useful as forensic evidence when using blow flies to estimate a minimum postmortem interval (mPMI).

Since high temperature has been implicated as a factor impacting blow fly physiology, we set out to quantify the high temperatures at which survival, egg deposition and egg hatch cease. We based our experimental design on the temperatures utilized by Prrish and Bickley [16] (37 °C, 44 °C) to investigate if the duration used by Ody et al. [18] (24 h) impacts survival and egg laying behavior. The number of trials completed was dictated by our limitations on human and material resources. Therefore, our other survival temperatures were chosen using a random number generator between 37 °C and 44 °C. It was our assumption that randomly selected treatment temperatures between 37 °C and 44 °C would still form a gradient through which survival at high temperatures can be observed and quantified. Using a range of temperatures including those used by Prrish and Bickley [16] was done to observe a gradient of survival at high temperatures. Regiment temperatures > 40 °C allowed us to observe egg laying and likelihood of hatch at temperatures beyond those used by Hans et al. [17] and Ody et al. [18]. Finally, we sought observe potential impacts of time in treatment by changing the oviposition substrate at the 12 h mark. The purpose of this was to analyze our observations for how duration under extreme heat stress impacts the likelihood of an egg deposition event. If *P. regina* and *L. sericata* have a temperature maximum for survival, then a UILT will be indicated before it is reached. If *P. regina* and *L. sericata* experience a thermally induced zone of resistance, then other biological processes, including oviposition, should diminish as the thermal maxima is approached.

## 2. Materials and Methods

The first objective of this study was to elucidate potential UILTs and an upper thermal maximum survival temperature of two forensically significant species in New Jersey, *L. sericata* and *P. regina*. The second objective investigated the upper thermal limits of oviposition of these species, a non-lethal but vital temperature threshold for blow fly colonization. Wild caught *P. regina* larvae were collected in April 2020 in New Brunswick, New Jersey (Latitude 40.48, Longitude −74.44) from a decomposing piglet (*Sus scrofa*) (Linneus) (Artiodactyla: Suidae) carcass. In July 2020 *L. sericata* were also obtained from New Brunswick, New Jersey using a yellow plastic funnel cone from a commercial fly trap secured to a Ziploc bag (Half Gallon Freezer, SC Johnson, Racine, WI, USA) (dimensions 26.8 cm × 14.3 cm × 8.8 cm) baited with ~50 g of fresh beef liver wrapped in a half paper towel. The trap was hung inside of a greenhouse which had ~10 missing panels, allowing flies easy access to the shelter located approximately 10 m from the pig carcass location.

### 2.1. Colony Rearing

Baited traps were used to continually supply the laboratory colonies for these studies with wild-type individuals [13] through Fall 2020. Identifications were made using the Jones et al. [22] identification key. Identification verification was made by co-author and American Board of Forensic Entomology (ABFE) certified member (M) Dr. Lauren M. Weidner, M-ABFE. Sex determination was done using the spacing of the eyes [23]. Six voucher specimens (3 female, 3 male) of each species were retained in repository at the Rutgers University Entomological Museum in New Brunswick, New Jersey. Bar codes for voucher specimens can be accessed digitally by entering the primary author’s name or this article’s DOI into the search query at: https://soar.libraries.rutgers.edu/ added on 30 July 2022. For more information, please see the “Data Availability Statement” below. *Phormia regina* adults used in this study were generations F_4_-F_8_. *Lucilia sericata* adults used in this study were generations F_3_-F_6_.

All adult colonies were kept in ‘BugDorm’ (MegaView Science Co., Ltd. Taiwan) rearing cages (dimensions 30 cm × 30 cm × 30 cm) lined with a paper towel. Adults were supplied granulated white sugar (Dinamo Foods Inc., Yonkers, New York, USA) and water in an Erlenmeyer flask (top secured with parafilm and a paper towel wick cut to approximately 1 cm × 6 cm) *ad libidum* [17]. To provide a protein source, adult flies were offered a Kim Wipe (Kimberly-Clark Professional™ Kimtech Science™ Kimwipes™ Delicate Task Wipers, 1-Ply, Roswell, Georgia, USA) saturated in fresh bovine blood, or a ~10 g piece of finely minced fresh bovine liver spread over a Kim Wipe (determined by availability of liquid blood) at approximately day 3 and 5 after eclosion for ~24 h. Colonies of adult flies were then offered the protein source approximately once a week until used in an experimental replicate. Colonies slated for experimental use were inspected every other day for deceased flies which were replaced with newly reared flies. Colonies to be used in replicates were offered a protein source for 24 h approximately 48 h before the start of the experiment. The protein source was also used to encourage spermatogenesis, vitellogenesis, and induce egg development [17]. Adults used in treatments were 7–14 days post eclosion. Colonies used in oviposition trials were protein fed until at least three females could be visually identified (in one viewing of approximately 5 min) as having pale distended ventral abdomens characteristic of fecundity.

Larval rearing containers were constructed using cylindrical clear quart-sized containers (Model L-5032/L-2532, PACTIV, Lake Forrest, IL, USA) secured with a tightly sealed lid retrofitted with breathable insect mesh secured with hot-glue to a window cut from the center. The bottom of the rearing chamber was lined with ~4 oz of white sand (Sandtastik Play Sand, Sandtastik Products, Ltd., Port Colborne, Ontario, Canada). A 9 oz plastic cup (ASQ950-20004) (SOLO Cup Company, Lake Forest, IL, USA) was trimmed to fit the rearing container and placed inside on top of the play sand [24]. This design allowed the feeding stage larvae to be reared in the plastic cup with the sand as a substrate to pupate. The plastic cup containing larvae was supplied with ~50 g of fresh beef liver which was replenished *ad libidum* to discourage nutrition as a limiting factor in development [18]. Pupae were removed from the sand lining the bottom of the rearing chambers via sifting and placed in individual 3.25 oz soufflé cups (B01MSBN1HX) (AV Inc., Guangzhou, Guangdong Province, China) secured with breathable lids. All pupae were kept on a laboratory bench, stacked in plastic mesh seedling trays (Medium Weight Mesh Tray, Johnny’s Seeds ID:7304.0, Dimensions: 10 ½” × 20 7/8”, Winslow, ME, USA) and observed daily for adult eclosion. A Microsoft Excel (Microsoft Corporation, Redmond, VA, USA) random number generator was used to randomize female and male adult flies into available fly cages. Temperature treatments were conducted in a Thermo Scientific Precision Model 818 Incubator (Thermo Fisher Scientific, Somerset, NJ, USA). The unit did not have humidity controls and was therefore supplemented with a mini humidifier (Pure Enrichment MistAire Travel—Ultrasonic Cool Mist Water Bottle Humidifier, Bear Down Brands, LLC, Santa Ana, CA, USA) and a two-gallon water bath in a plastic bin. A HOBO data logger (HOBO^®^, Onset^®^ Computer Corporation, Bourne, MA, USA) was used to record temperature and relative humidity. Each replicate in both objectives was treated with a 16:8 Light/Dark cycle.

### 2.2. High Temperature Survival Trials

Each experimental colony used in replicates consisted of 20 males and 20 females (adults) in a cubic mesh cage lined with a paper towel [18]. Each replicate consisted of three colonies exposed to a given temperature treatment (see below) for a 24 h period. Incubators were room temperature (~20 °C) when colonies were added and then set to the target temperature. This allowed for an approximately a 15 min temperature acclimation period as the internal temperature increased. This was done assuming protective mechanisms such as Heat Shock Proteins would be upregulated. 

After each trial, all treated individuals were allowed to rest in their cages on the laboratory bench for 24 h to allow any knocked-down flies time to recover [25]. Survival counts were only taken after the 24 h post-experiment rest period at room temperature. Flies that displayed no movement when perturbed with mechanical stimulus (first agitating the cage, then probing individual flies) were considered dead. Flies that displayed any movement (i.e., moving of legs or wings) were counted as survived. Flies were then counted, sex determined, and euthanized by freezing before being discarded. One control replicate (Replicate 0, three colonies) for each species was completed by observing the number of flies surviving 24 h on the laboratory bench at ~22 °C. Survival trials were completed at temperatures of 37 °C, 41 °C, 42 °C, and 44 °C. Survival for all control colonies was near 100%, and these data were not used in the analysis. 

### 2.3. Oviposition Trials

Data from survival trials were used to inform temperature regimens in oviposition trials. Oviposition trials quantified if egg deposition occurred, the quantity of eggs deposited, and if any of eggs laid during treatment were capable of hatching. Experimental setup for oviposition trials was identical to survival trials with the addition of a 50 g (+/−6 g) piece of beef liver that was replaced at the ~12 h mark. Replacing the oviposition substrate was done to count the number of eggs, assuming this period would be before hatch, and to quantify oviposition through the duration of the replicate. Prior to oviposition trials beef liver was placed on half a Kim Wipe on a Petri dish and frozen in the laboratory freezer to preserve freshness. The first (“12 h”) oviposition substrate (Petri dish) offered to each experimental colony was still frozen. It was assumed this would impede availability until the incubators’ internal target treatment temperature was reached. The substrate for the second half (“24 h”) of the experiment was allowed to thaw on the bench until the time of replacement. Our assumption was the cooler oviposition substrate was conducive to egg laying. Therefore, successful egg deposition would be a factor of the adult’s ability to oviposit, reducing the likelihood that an unacceptable substrate would confound observations.

Each time an oviposition substrate was removed from a colony it was examined under a dissecting light microscope for eggs. Substrate that did not contain eggs or maggots at the time of harvest was immediately discarded after inspection. The number of eggs were manually counted using a clicker-counter. Eggs in large clutches (i.e., an inability to visualize the number eggs at the center of the cluster) were gently teased apart with a dampened paintbrush to ensure all eggs were counted. If live maggots were present at the initial inspection, the total number of eggs (hatched and unhatched) and larvae were recorded. All substrate containing eggs was kept on the bench for five days and inspected daily to quantify hatching success. A determination of “Eggs, No hatch” or “Eggs, Yes Hatch” was made for each replicate colony’s substrate containing eggs until the fifth day. If no hatch was observed by day five, a determination of “No Hatch” was made and the substrate discarded. No control colonies were used for oviposition experiments because temperatures were determined from preliminary analysis of the survival experiment. Oviposition trials were completed at temperatures of 40 °C, 42 °C and 43 °C for both species. An oviposition trial was completed for *P. regina* at 41 °C after an incubator programming error resulted in a non-target temperature replicate. 

### 2.4. Analysis

All statistical analyses were done in R version 4.1.1 [26]. Plots generated through R were edited in Adobe Illustrator. General Linear Models (GLM) were used to analyze the effect of treatment temperature on adult survival, egg hatch, and live larvae. A model was developed to compare the probabilities of survival of the two species in our study, *L. sericata* and *P. regina*. Our survival model tested species and temperature regimen for a set duration of 24 h. Covariates within the survival model were sex, replicate, and replicate colony. The survival model was used to test the hypothesis that a UILT will exist for both species and will delineate a zone of resistance between the UILT and LT_Max_. A GLM plot was also used to visualize the distribution in the number of eggs laid.

Preliminary data from survival analysis was used to elucidate temperature regimens likely within both species zone of resistance to observe its impact on oviposition. Models describing oviposition and progeny of adult flies used species and temperature regimen as factors. In these models, three sets of binomial covariates were measured; check/duration (0 = 12 h, 1 = 24 h), egg presence (0 = no, 1 = yes), and egg hatch (0 = no, 1 = yes).

Temperature and humidity data collected by digital datalogger was uploaded via Microsoft Excel and averaged in Microsoft Excel for survival trials. We did not include the abiotic factor of humidity in our analysis models because humidity could not be controlled precisely in the model of incubator used for this experiment. During oviposition trials, temperature readings on the dataloggers were noted at the 12 h check when oviposition substrate was switched. Recordings from the datalogger indicated a significant disruption to internal temperature at the 12 h check. This was caused by having to keep the incubator door open long enough to switch the oviposition substrate in all three colonies per replicate. This complicated a straightforward determination of average internal incubator temperature. Therefore, the internal temperature displayed on the datalogger screen was recorded when the incubator door was first opened for the 12 h check. This temperature was considered valid if the temperature displayed on the datalogger screen at the end of the experiment was +/−0.5 °C of the 12 h reading. A wide latitude was used because the incubator unit being used was prone to fluctuations in temperature over 40 °C. 

## 3. Results

**Figure 1:**
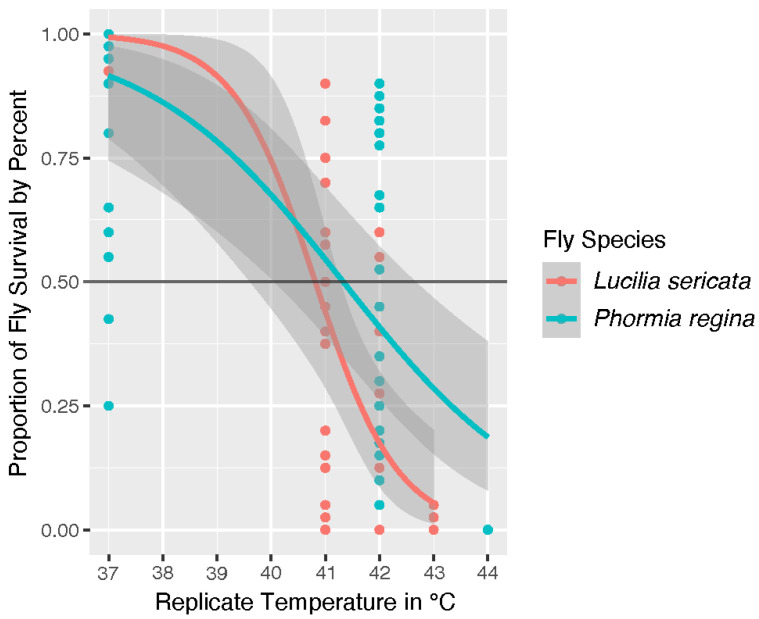
Survival of *Lucilia sericata* (light red) and *Phormia regina* (teal) at 37 °C, 41 °C, 42 °C, 43 °C and 44 °C. Temperature has a significant impact with a *p* = 6.10 × 10^−8^ while species does not with a *p* = 0.096. The grey area around the trend lines denotes the margin of error. A black bar at the 50% survival mark is to visualize the model-projected Upper Incipient Lethal Temperature (UILT) for both species.

Our statistical analysis evaluated the *p*-value (“*p*”) where we set the alpha value to 0.05 as the delineation to indicate whether the parameters from the GLM statistically differed (“significant”) from the experimental results we collected. None of our analyses determined replicate or replicate colony to be statistically significant factors in modeled outcomes. The survival of *L. sericata* and *P. regina* adults at different temperatures is shown on the graph in Figure 1. The trendline represents the model’s prediction of survival with the gray area surrounding the trendline representing the margin of error. Our model predicts *L. sericata* will reach the UILT just under 41 °C while *P. regina* is predicted to reach this threshold just above 41 °C. However, temperature was determined to be a significant factor with a *p* = 6.10 × 10^−8^ while species was not with a *p* = 0.096. 

**Figure 2:**
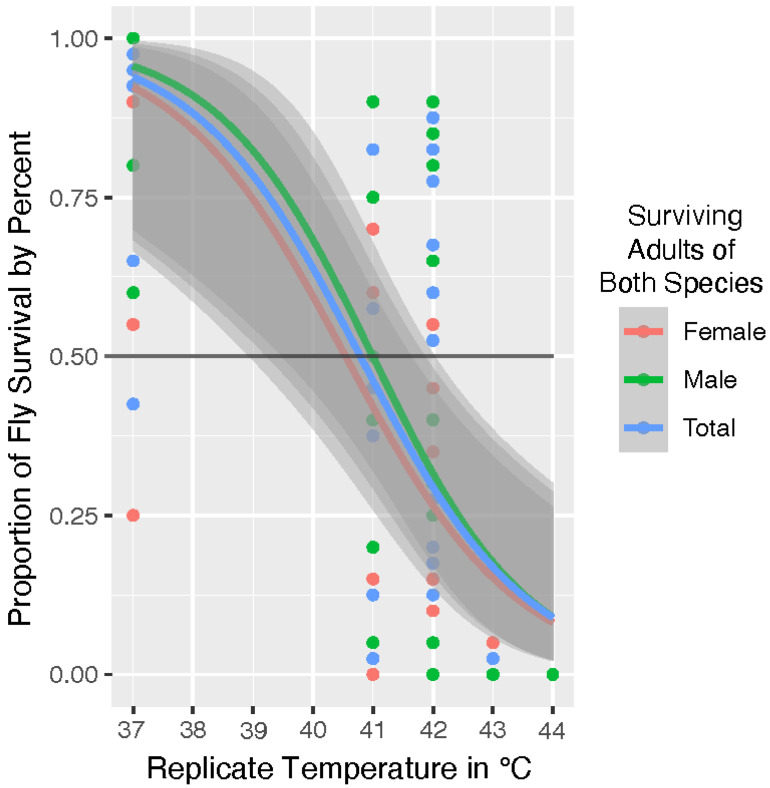
Survival analysis of female (red) and male (green) adult flies across both species compared to the total (blue) proportion of surviving flies of *Lucilia sericata* and *Phormia regina*. Model analysis of both species indicates an Upper Incipient Lethal Temperature (UILT) for both species at 41 °C. Sex was not significant (*p* = 0.542) in survival while males survived slightly better than average.

The overlapping margin of error shown in the species graph of Figure 1 is further illustrated in the total fly analysis shown in Figure 2. We took a comparative approach to our analysis. Therefore, once our model determined that species was not a statistically significant factor in survival, we analyzed adult blow fly survival by sex across both populations. The Figure 2 graph includes trendlines for the covariates of sex. Adult male flies survived slightly above average but sex was not determined to be significant (*p* = 0.542) in survival. Our model indicates the overall UILT of both species is 41 °C, with a zone of resistance that extends from ~41.0 °C to the LT_Max_ at ~44 °C.

**Figure 3:**
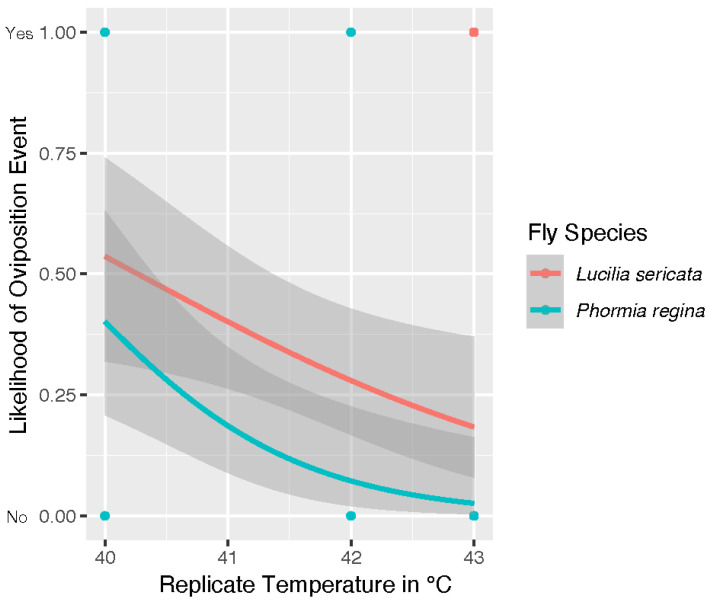
Model projected likelihood of oviposition event for *Lucilia sericata* (light red) *and Phormia regina* (teal) as temperatures increased measured from data collected at 40 °C, 42 °C, and 43 °C for both species. Outcomes were measured as yes (eggs) or no (no eggs). These data indicates Lucilia sericata is statistically more likely (*p* = 0.034) to lay eggs at temperatures over 40 °C. Temperature was also a significant factor with a *p* = 3.01 × 10^−4^.

**Figure 4:**
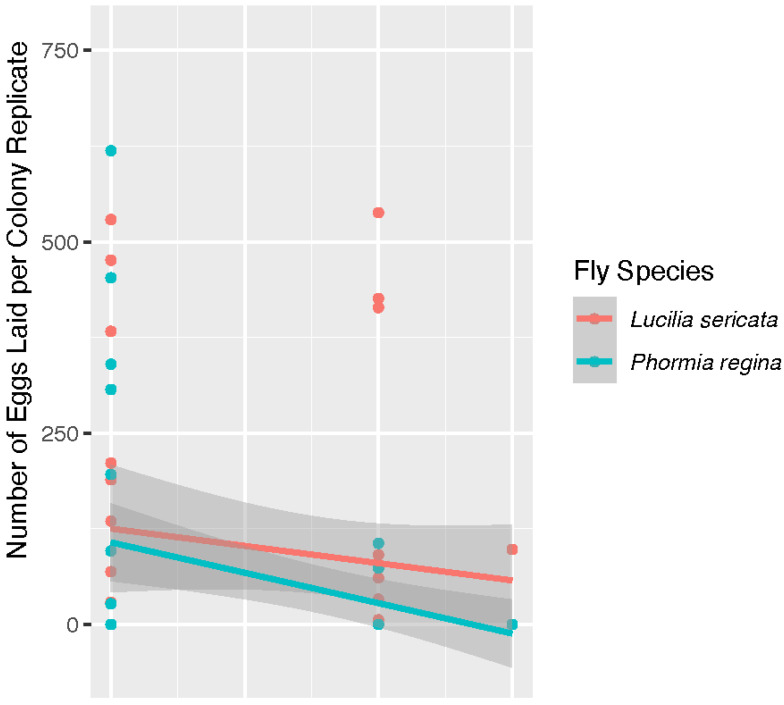
A plot of the number of eggs laid by *Lucilia sericata* (light red) and *Phormia regina* (teal) at temperatures at 40 °C, 42 °C, and 43 °C. In this analysis, fly species was not a significant factor with a *p* = 0.112 while temperature was significant with a *p* = 0.009.

Figure 3 is our model for the likelihood of egg deposition, which was a binomial choice. In this model, species was significant (*p* = 0.034) with *L. sericata* more likely to lay eggs at temperatures over 40 °C. The model in Figure 3 analyzed the egg data collected for both species (40 °C, 42 °C, and 43 °C). As stated above, only *P. regina* was treated at 41 °C in oviposition trials. *Phormia regina* laid eggs in two of the three replicate colonies producing 43 and 644 eggs, respectively, observed at the 12 h check. A distribution of the number of eggs laid for adults of both species is shown in Figure 4. In this analysis, species was not determined to be a significant factor (*p* = 0.112) in the number of eggs laid by colony when oviposition events did occur. The *P. regina* 41 °C egg data is included in the analysis for egg distribution in Figure 4. The 41 °C *P. regina* colony which deposited 644 eggs also produced 2 live larvae. Analysis of all live larvae observed for the duration of the study is reported below.

**Figure 5:**
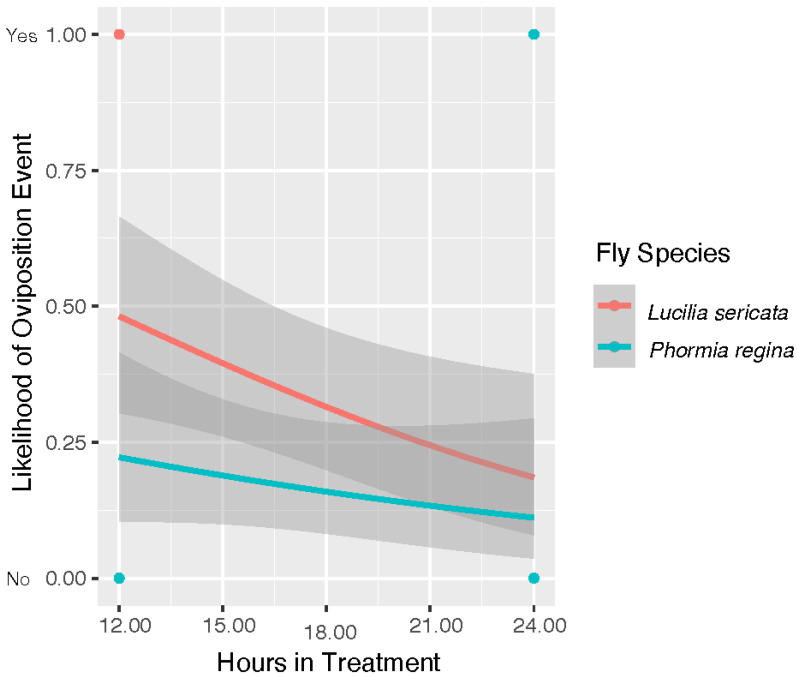
A plot modeling the likelihood of an oviposition event (yes/no) as time in treatment progresses. The *y*-axis shows the 12 h and 24 h checks when a determination of yes/no was made for oviposition events. These data indicate that both species are less likely to oviposit post 12 h in treatment at 40 °C, 42 °C, and 43 °C. In this analysis temperature (*p* = 2.26 × 10^−4^), hour (*p* = 0.009) and fly species (*p* = 0.028) were all determined to be significant factors in likelihood of oviposition as time in treatment progressed.

A model projecting the likelihood of egg deposition over time spent in treatment is shown in Figure 5. In this analysis, both egg deposition and hour are binomial choices representing eggs/no eggs and first/second check, respectively. This analysis has indicated all three factors of temperature (*p* = 2.26 × 10^−4^), hour (*p* = 0.009) and fly species (*p* = 0.028) were significant. This model shows a trend toward decreasing likelihood of egg deposition for both species as time in treatment progressed. Temperature was also significant factor in likelihood of egg hatch as a yes/no event (model code in R file). In our experiment, we observed egg hatch from two colonies of *L. sericata* that oviposited in 43 °C trials. However, our statistical model indicates that that neither hour (*p* = 0.993) nor species (*p* = 0.062) was likely significant.

The total number of live larvae observed at their respective treatment temperatures across both time checks was analyzed (model code in R file). This data includes the two live larvae observed for *P. regina* in the 41 °C trials. In this analysis, our model projects species will not be a significant factor (*p* = 0.255) in the likelihood of live larvae being observed on an oviposition substrate ≤ 12 h post-presentation at temperatures ≥ 40 °C. This model also does not identify temperature as a significant factor (*p* = 0.096). 

## 4. Discussion

The survival results found in our study are consistent with data reported by Prrish & Bickley [16] where Mid-Atlantic populations of *Phormia regina* reached a LT_max_ by 44 °C. Our experiment built on this work by treating all colonies for a 24 h period, replicating the treatments, and utilizing a standard protocol across treatments and species with similar results were observed for *L. sericata*. Checking for survival only after a 24 h rest period may be a confounding factor as some flies may have perished from factors unrelated to treatment. Future experiments may seek to elucidate the proportion of flies that are likely to die in the subsequent hours after high temperature treatment. Our minimum (37 °C) and maximum (44 °C) experiment trial temperatures were chosen to reflect the two temperatures used by Prrish and Bickley [16] to study *P. regina* longevity at high temperatures. A preliminary data analysis indicated both species entered the zone of resistance ~41 °C and reached 0% survival by 44 °C. To observe egg laying within the zone of resistance of blow flies, we used 42 °C and 43 °C, with a minimum of 40 °C to reflect the maximum temperatures utilized by Hans et al. [17] and Ody et al. [18]. While Prrish and Bickley [16] observed egg deposition and egg quantity at 37 °C and 44 °C, their oviposition substrates were presented to flies post-treatment and after a rest period. 

Both species produced eggs that hatched at 40 °C while only *L. sericata* was observed to regularly lay eggs that hatched above that temperature. However, eggs laid by both species within our indicated zone of resistance were often unsuccessful at hatching. Notably *P. regina* ceased egg laying at its zone of resistance threshold. Colonies of *L. sericata* were more likely to exhibit an egg deposition event than *P. regina* even though both species showed a decline through the zone of resistance. We recorded diminishing quantities of eggs laid as temperature increased. Our observations are consistent with the trend of diminishing eggs deposited reported by Hans et al. [17], who reported *P. regina* deposited more eggs than *L. sericata* as treatment temperature increased until 40 °C. We observed more eggs deposited by *L. sericata* > 40 °C. However, we believe our observations are broadly consistent with those reported by Hans et al. [17] because we assume biogeographical factors (i.e.,: seasonal climate, coastal proximity) could impose different selection pressures on blow flies from different regions (i.e.,: Ontario, Canada versus New Jersey, USA). In New Jersey, *P. regina* was shown to be most abundant during the autumn season across the state [24]. The same survey found that *L. sericata* was the dominant species for all seasons except winter for traps set in the same location our flies came from. The dominance of *L. sericata* in warm seasons may be driven by a mechanism to continue laying eggs during periods of extreme heat stress.

Our statistical model projects that the studied population of *L. sericata* is probably capable of exhibiting an egg deposition event for a longer duration in treatment compared to *P. regina* from the same region. Observations from our study indicate both species deposit fewer eggs after 12 h in extreme heat. Further, eggs laid in the second half of the oviposition trial were the least likely to hatch. Live first stage larvae produced by *L. sericata* at 40 °C and 42 °C within the first 12 h of being presented with an oviposition substrate was an unexpected result. *Phormia regina* produced live first stage larvae within 12 h of first being presented with an oviposition substrate at least once at 40 °C and 41 °C each. Data reported from earlier experiments on lifecycle developmental rate at high temperatures of *Protophormia terraenovae* (Robineau-Desvoidy) (Diptera: Calliphoridae) indicated egg hatch may occur as soon as ~9 h after oviposition [27]. An important caveat for our experiment is that the first oviposition substrate presented in each replicate was given still frozen to impede immediate access. It is unknown if the instances we observed of egg deposition post 12 h were from a single/repeated female or conspecific females. A single female laying eggs multiple times over a 24 h period at >40 °C may indicate the retention of individual-level plasticity in challenging environments, or a potential physiological survival response. Our observations on the impacts of heat and time are significant from the perspective of utilizing *L. sericata* and *P. regina* to determine Time of Colonization (TOC) or a mPMI. These data indicate a heatwave lasting 12 h or longer has the potential to augment estimates of TOC or mPMI by as little as 0.5 days to more than 1.0 days.

While the temperatures and duration of replicates may seem extreme, we sought to determine the limits of survival and egg deposition. Further, from 25 June 2021 to 7 July 2021 the coastal Northwestern portion of North America experienced an extreme weather event known as a “heat dome”. Heat domes are complex atmospheric systems that require low wind and can be driven by pollution associated with urban/suburban sprawl [28,29]. During the 2021 Western North America heat wave the Canadian Government reported Lytton, British Columbia reached a high temperature of 49.6 °C on 29 June 2021. The heat dome was attributed to 740 additional human deaths in British Columbia compared to the same period in the previous year [30]. Data from Wireless Fidelity (Wi-Fi) connected thermostats indicated some internal home temperatures were around 40 °C during the heat wave [30]. The design of our experiment, where blow flies were restricted to an insect cage, may be most analogous to potential forensic investigations where adult flies may become trapped in enclosed automobiles and dwellings when seeking carrion. 

Carrion concealed or within a confined space can present a barrier to primary colonization by calliphorids. Field trials of *Sus scrofa* concealed in automobile trunks in Louisiana, USA and Vancouver, Canada demonstrated delayed colonization by 3 days and 3–6 days, respectively [5,31]. Australian field trials of *S. scrofa* in the car driver’s seat demonstrated delayed colonization by ~1 day [32]. An associated experiment modeled the impact of solar radiation on the temperature difference of internal car cabins versus the outer environment. This investigation found even with vehicle color accounted for, sealed cabin temperatures can dramatically exceed the environmental ones [33]. The Australian and Canadian experiments both noted accelerated decomposition catalyzed by higher temperatures within the cars, compared to outer ambient temperatures [5,31]. The ratio of pupae to adult flies remaining trapped within the vehicle led Malainey & Anderson [31] to conclude the adult flies were likely incapable of egress from the space. This demonstrates situations where adult flies may become trapped within the high temperature arena.

An indoor study of three *S. scrofa* carcasses in separate rooms of the same suburban house in Edmonton, Alberta demonstrated colonization was delayed up to five days and decomposition was slowed compared to outdoor carcasses [34]. The accelerated decomposition exhibited in car trunks reported by Malainey & Anderson [31] is likely an effect of the additional heat retained by the car over a longer period versus more spacious rooms in a dwelling. Modeling used to predict sealed cabin temperatures indicate that a 30 °C day may raise internal temperatures beyond our observed limits of survival and oviposition [34]. When forming the predictive models, the scenario of a car window being opened by 2.5–5 cm was also investigated. Minimal ventilation of the car cabin was shown to raise internal temperatures still dramatically beyond ambient. Our results indicate an extreme heatwave lasting over 12 h may further augment time of colonization through conditions inconducive to oviposition. Thermostat data from some Canadian homes during the 2021 Western North America heat wave indicate internal home temperatures may reach oviposition-augmenting temperatures in extreme heat events [29]. This presents three potential scenarios for such remains during extreme heat events. First, that remains within a suburban home may experience an even more prolonged time of colonization than observed by Anderson [34] when temperatures exceed the maxima range of oviposition. Second, if colonization has already occurred by the start of the extreme heat event, internal room temperatures may catalyze the accelerated rate of decomposition observed by Malainey & Anderson [31]. Finally, carrion accessible through an open car window may be rendered “unavailable”. Solar radiation can create internal cabin temperatures incompatible with survival and/or oviposition consistent with modeling demonstrated by Dadour et al. [33]. In this third situation, colonization is delayed by the adult fly egressing from scene and/or depositing eggs unlikely to hatch. This is similar to our survival trials where escaped flies survived while adult flies trapped within the arena perished. In these instances further replicates were done. 

In a temperature-controlled pitfall trap building experiment of larval *Myrmeleon bore* (Tjeder) (Neuroptera: Myrmeleontidae), larvae in warmer treatments consistently built larger pitfall traps compared to control animals [35]. The Antoł et al. [35] results indicate that insects can modify behaviors in response to high temperatures. The impacts of both high temperatures and periods of lower humidity may have been mitigated by the water supplied via Erlenmeyer flask and the supplemental water bath. Our analysis focused on the abiotic factor of temperature and humidity was not included in our analysis. This is a potentially confounding factor of our observations. The model of incubator used in our experiment did not have humidity controls. Humidity data showed some trials averaged 70% relative humidity, which may indicate significant differences in humidity within and across replicates. The impact of humidity on blow flies at temperatures > 40 °C requires further investigation because our experimental design cannot account for how humidity may have impacted our results. This will require using an incubator unit capable of maintaining and monitoring internal humidity. In high temperature survival and knockdown experiments using *C. rufifacies* and *C. macellaria*, the availability of water was found to increase the likelihood of survival [20,21]. While not recorded, a visual inspection of our flies as they were removed from incubators indicated that surviving flies would often perch on the water source. This may have been a behavioral strategy to find a microclimate as a refuge. Available water may have also been used for “bubbling” behavior which allows for evaporative heat loss. In a high temperature survival investigation of the blow fly *Calliphora vicina* (Robineau-Desvoidy) (syn. *C. erythocephala*) (Diptera: Calliphoridae), adults confined to small test tubes had a UILT between 40.5 °C and 41.13 °C. While consistent with our results, the Davison [36] UILT temperatures were slightly lower. This may indicate behavioral inhibition may be a confounding factor in some investigations, or that there is variability across species and populations. 

An experiment of larval *Musca domestica* (Linneus) (Diptera: Muscidae) at 42 °C found that relative expression of the gene that codes of Heat Shock Protein (HSP) HSP70 was significantly upregulated after 15 min in treatment [37]. In a high temperature experiment of adult fruit moths *Grapholita molesta* (Busck) (Lepidoptera: Tortricidae) it was found that HSP Gmhsp90 and Gmhsp70 were both upregulated [38]. Treatment durations of ~1 h showed maximum expression of both HSP. With durations of ≥1.5 h showing lower upregulation of both HSP but still considerably higher than 0 min [38]. This trend indicates treatment duration is negatively correlated to HSP expression. The survival rates observed in our experiment are likely an impact of our treatment duration. If the trend found in adult *G. molesta* can be applied to our experiment, then the duration of 24 h may have overwhelmed the ability of the organism systems to compensate with protein chaperoning. Future research may seek to quantify HSP expression in *P. regina* and *L. sericata* as high temperature treatment duration approaches 24 h. Measuring the expression of adult blow fly HSP in the successive hours post-treatment is another potential line of inquiry.

Being poikilotherms, the temperatures of blow flies are largely controlled by ambient temperatures. Without other dedicated pathways, poikilothermic animals must largely rely on behavioral thermoregulation and/or molecular mechanisms such as HSP to regulate body temperature. Therefore, one assumption may be that ambient temperatures are reflective of the core body temperature of adult flies in high temperature treatment. If accurate, our experiments indicating blow flies likely enter the zone of resistance by 41.5 °C and reach LT_max_ between 43 °C–44 °C may be consistent with rodent models. In a review of thermal biology work performed on the laboratory rat *Rattus norvegicus* (Berkenhout) (Rodentia: Muridae), Gordon [39] reported that body size and sex impacted rate of survival at high temperatures on an individual level. However, trends from experiments indicated *R. norvegicus* generally reached LT_max_ when internal core body temperatures reached 42.5 °C [39]. With further investigation to elucidate what potential patterns may exist, it may be possible to use the poikilothermic properties of some insects (i.e., blow flies) to model the physiological processes of higher vertebrates when internal body core temperatures are within the zone of resistance and approaching LT_max_. Weather events and climate change are two conditions that have been implicated in driving cycles of infectious disease in humans [40]. Dipterans are considered an acceptable model to study drivers of human disease because flies share conserved molecular pathways with humans [41]. Using blow flies to study mechanisms like HSP at temperatures where *R. norvegicus* reaches LT_max_ may be a more accessible alternative to experimentation on rodents for some researchers investigating climate-related disease drivers. If demonstrated, this could expand the use of blow flies as models to study the conserved molecular pathways activated to mitigate heat stress which also occur in humans.

## 5. Conclusions

Our experiments demonstrated that survival diminishes for the New Jersey populations of blow flies as temperatures approach 44 °C, which is likely the thermal limit of survival for *Phormia regina* and *Lucilia sericata*. The exploitation of microclimates may contribute to the ability of an individual fly to survive. *Phormia regina* was able to lay eggs with egg hatch up to 41 °C, with 43 °C being the limit of *L. sericata* successful egg deposition. This indicates *P. regina* has a sub-lethal range of temperatures where successful egg deposition is unlikely. *Lucilia sericata* exhibited a narrower range of sub-lethal temperatures where egg laying did not occur. These observations indicate that sublethal temperatures may delay or cease completion of the reproductive cycle. This would potentially augment estimates of mPMI and TOC. Finally, the presence of unviable blow fly eggs in confined spaces may indicate environmental conditions which were incompatible for hatch. The statistically similar number of eggs deposited at high temperatures by *P. regina* (a cool tolerant species) and *L. sericata* (a warm season preferring species) have implications for decomposition ecology to be impacted by the projected rising temperatures associated with climate change.

## Data Availability

Data from this experiment is permanently stored at RUResearch, the data portal of RUCore, the Rutgers Community Repository to preserve scholarly output generated at the University. The R program code and associated data files can be accessed by entering the DOI and/or article title into the search query box at: https://rucore.libraries.rutgers.edu/research/ (accessed on 24 August 2022).
